# Rectosigmoid Intussusception Presenting as Rectal Prolapse Secondary to Adenocarcinoma in a Young Postpartum Woman: A Report of a Rare Case

**DOI:** 10.7759/cureus.93469

**Published:** 2025-09-29

**Authors:** Mohammed A Alsuhaimi, Mohmmed A AlHewishel, May S AlKhaldi, Abdurahman AlSumaihi

**Affiliations:** 1 Surgery, Dammam Medical Complex, Dammam, SAU; 2 General Surgery, Dammam Medical Complex, Dammam, SAU

**Keywords:** case report, colorectal adenocarcinoma, laparoscopic low anterior resection, postpartum, rectal prolapse, rectosigmoid intussusception, sigmoid colon cancer

## Abstract

Rectal prolapse and intussusception are rare in adults and particularly unusual in the postpartum period. We present the case of a 39-year-old woman, one month postpartum following cesarean delivery of twins, who developed rectal prolapse during pregnancy associated with persistent constipation. She later presented with rectal bleeding and mucus discharge. Computed tomography of the abdomen and pelvis demonstrated rectosigmoid intussusception, while colonoscopy revealed a large fungating and friable mass at 25 cm from the anal verge. Biopsies confirmed adenocarcinoma. Magnetic resonance imaging demonstrated a 7.5 cm sigmoid lesion confined to the bowel wall without extramural extension or suspicious lymph nodes. The patient underwent laparoscopic low anterior resection with stapled colorectal anastomosis. Pathology confirmed moderately differentiated adenocarcinoma invading the muscularis propria with clear resection margins and 17 negative lymph nodes (pT2N0, Stage I). Her recovery was uneventful, and she remains well on follow-up. This case highlights the importance of considering malignancy in young adults presenting with persistent rectal prolapse or intussusception, particularly in the postpartum setting, and demonstrates the role of timely imaging and colonoscopy in establishing the diagnosis.

## Introduction

Intussusception is defined as the telescoping of one segment of the intestine into another and is a common cause of bowel obstruction in children. In adults, however, it is rare, accounting for less than 5% of all intussusceptions and fewer than 1% of intestinal obstructions [1]. Unlike pediatric cases, which are frequently idiopathic, more than 70% of adult intussusceptions have a pathological lead point, with malignancy being the predominant etiology in colonic lesions [2].

Rectal prolapse in adults is typically considered a benign condition, often associated with chronic constipation, multiparity, or pelvic floor weakness. Its occurrence during pregnancy or in the postpartum period is rare and usually attributed to physiologic changes, straining, and increased intra-abdominal pressure [3]. Colorectal carcinoma presenting initially as rectal prolapse or intussusception in a young postpartum patient is exceedingly uncommon, making diagnosis challenging and sometimes delayed [4].

We present the case of a 39-year-old postpartum woman who developed rectosigmoid intussusception secondary to adenocarcinoma, initially manifesting as rectal prolapse during pregnancy. This report highlights the importance of maintaining a high index of suspicion for malignancy in atypical or persistent prolapse presentations, even in young patients, and underscores the role of cross-sectional imaging and colonoscopy in timely diagnosis and management.

## Case presentation

A 39-year-old healthy woman, medically and surgically free, one month postpartum following twin vaginal delivery, presented to the emergency department with rectal prolapse associated with bleeding per rectum and mucus discharge. She reported that her symptoms began approximately one month after delivery, when she first experienced rectal prolapse with painful defecation and persistent constipation requiring multiple laxatives with minimal relief. Notably, she had suffered from constipation since the second trimester of pregnancy, which persisted postpartum. Since the onset of prolapse, her symptoms had progressively worsened, prompting her emergency department visit. She denied fever, abdominal pain, vomiting, or urinary complaints.

On presentation, she was conscious, alert, and vitally stable. Abdominal examination revealed a soft, non-tender abdomen. Per rectal examination demonstrated a full-thickness, circumferential rectal prolapse measuring approximately 5-6 cm in length, with blood-stained mucus. The prolapsed bowel appeared mildly congested but not severely oedematous, and there was no associated anal fissure. Laboratory investigations demonstrated a hemoglobin of 11.8 g/dL (baseline ~10 g/dL) and a white blood cell count of 8.34 ×10⁹/L. Tumor markers were within normal limits, with CA 19-9 at 15.62 U/mL and CEA at 0.0 ng/mL (Table 1).

**Table 1:** Laboratory investigations and reference ranges AST: aspartate aminotransferase; ALT: alanine transaminase; CA: carbohydrate/cancer antigen; CEA: carcinoembryonic antigen

Parameters	Patient Value	Normal Range
Hemoglobin	11.8 (baseline ~10)	12–16
White Blood Cells (×10⁹/L)	8.34	4.0–11.0
CA 19-9 (U/mL)	15.62	< 37
CEA (ng/mL)	0.0	< 3 (non-smoker), < 5 (smoker)
Creatinine (mg/dL)	0.9	0.6–1.2
Urea (mg/dL)	28	10–45
AST (U/L)	22	< 40
ALT (U/L)	24	< 40
Total Bilirubin (mg/dL)	0.8	0.2–1.2

The prolapse was manually reduced in the emergency department using lubricated gauze and gentle pressure while the patient was placed in the left lateral position. Bowel preparation was achieved with polyethylene glycol solution, and colonoscopy was performed electively during admission. 

**Figure 1:**
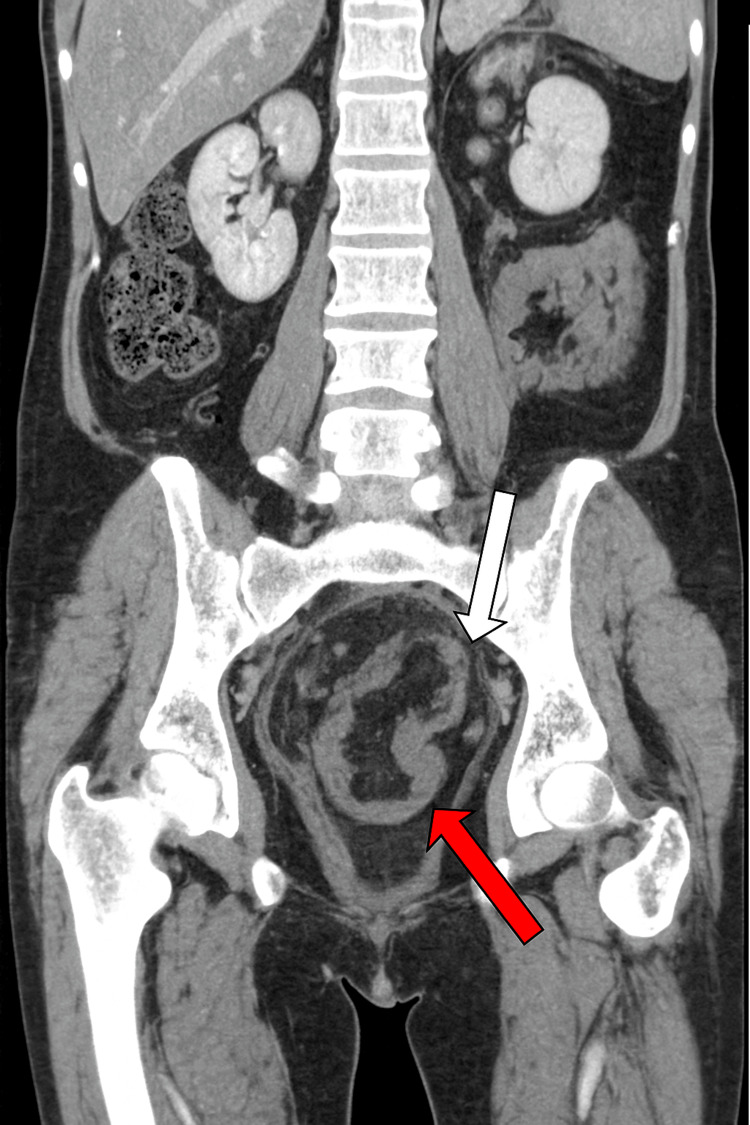
Contrast-enhanced CT (coronal view) showing rectosigmoid intussusception with the classic “target sign.” The red arrow highlights the intussusceptum (telescoping inner bowel), and the white arrow highlights the intussuscipiens (receiving outer bowel).

**Figure 2:**
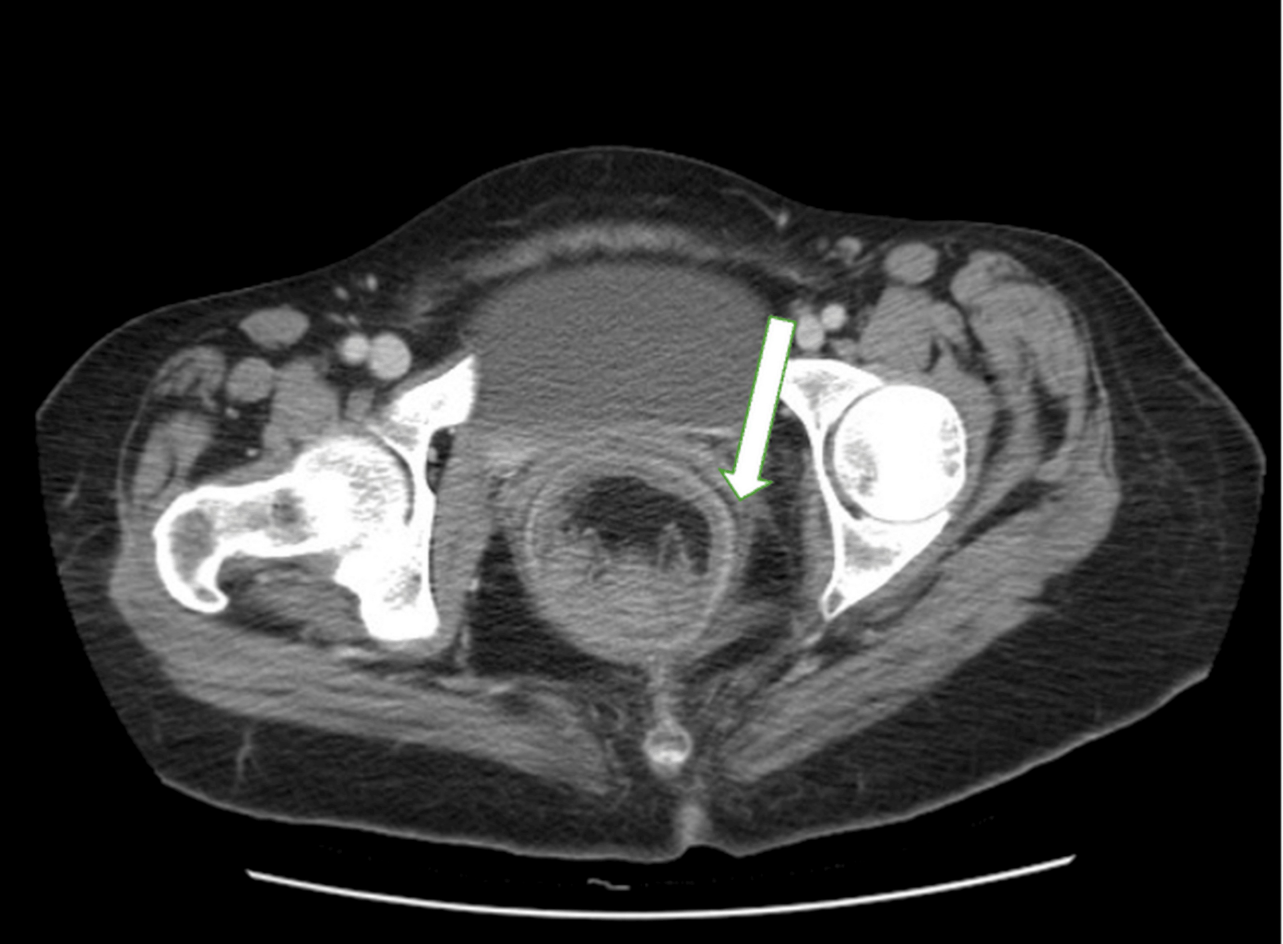
CT abdomen and pelvis, axial view, showing the classic “target sign” of rectosigmoid intussusception (arrow)

**Figure 3:**
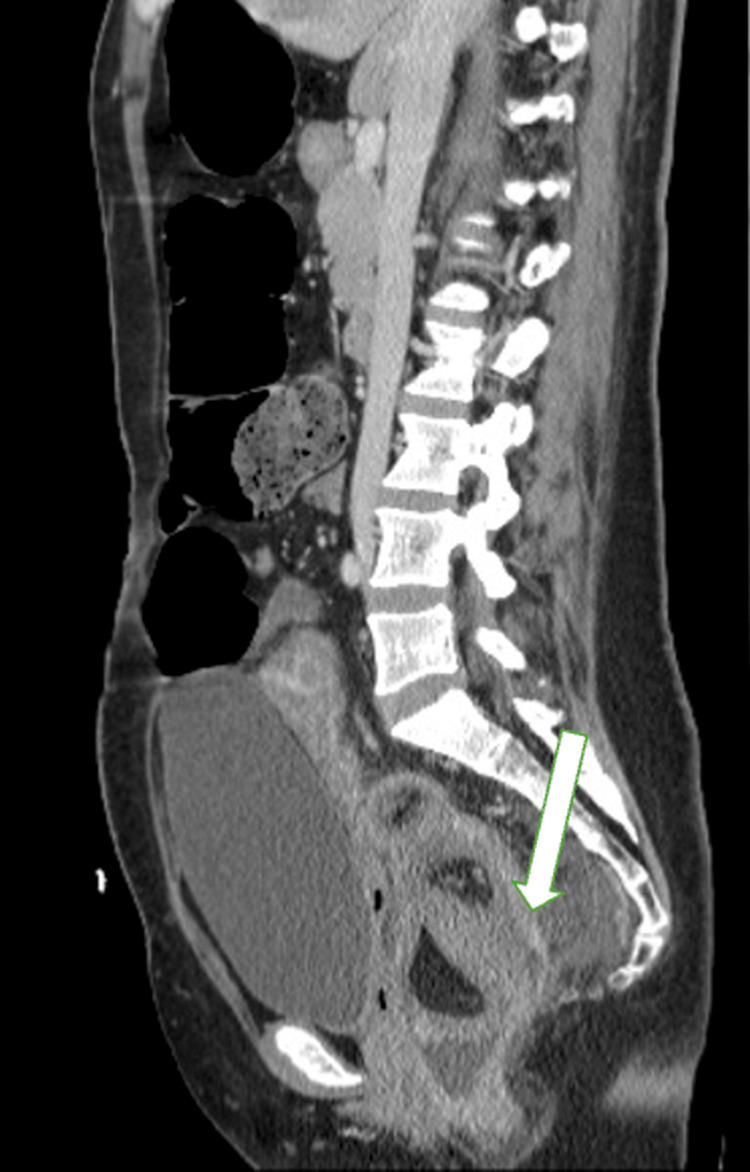
: CT abdomen and pelvis, sagittal view, demonstrating rectosigmoid intussusception (arrow).

A contrast-enhanced CT scan of the abdomen and pelvis demonstrated rectosigmoid intussusception, visualized as a bowel-within-bowel configuration consistent with the “target sign”, spanning approximately 14 cm of the rectosigmoid colon. The lead point appeared to be at the junction of the sigmoid and descending colon. The proximal large bowel was prominently dilated, measuring up to 5.8 cm. Surrounding inflammatory changes were noted, without frank obstruction (Figures 1-3). 

Colonoscopy, performed electively after bowel preparation with polyethylene glycol solution, revealed a large (>4 cm) fungating mass located approximately 25 cm from the anal verge. The lesion was friable, bled easily on contact, and caused near-complete luminal obstruction, preventing passage of the colonoscope beyond the mass. Multiple biopsies were obtained, although technically difficult due to friability. No synchronous polyps were visualized in the distal colon, and there was no evidence of intussusception during the procedure. Histopathology confirmed a moderately differentiated adenocarcinoma. A staging chest and pelvic CT demonstrated no evidence of metastatic disease.

**Figure 4:**
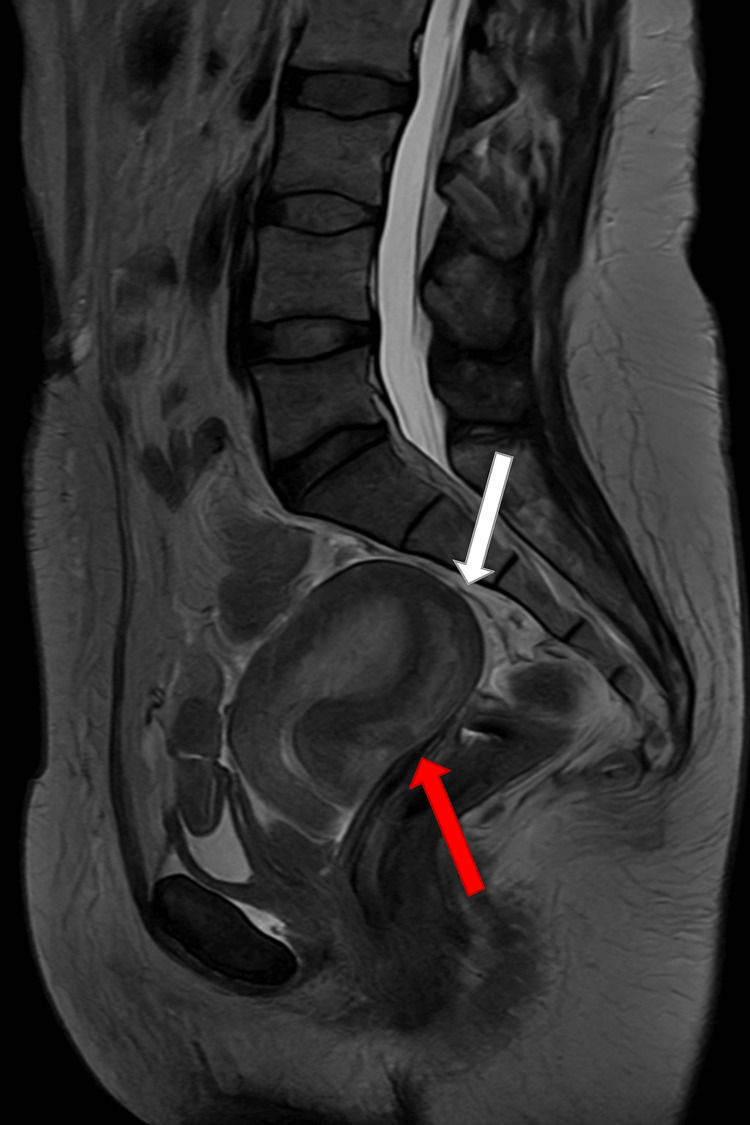
Pelvic MRI (sagittal view) demonstrating rectosigmoid intussusception The bowel-within-bowel configuration is shown. The red arrow highlights the intussusceptum (inner telescoping bowel loop), while the white arrow indicates the intussuscipiens (outer receiving bowel loop).

Pelvic MRI, obtained the week following colonoscopy, showed an interval reduction of the intussusception. A 7.5 cm × 1.5 cm asymmetric sigmoid wall thickening was identified, confined to the bowel wall without evidence of extramural invasion. No suspicious lymph nodes or distant disease were seen. Trace pelvic free fluid was also noted (Figure 4).

The patient underwent laparoscopic low anterior resection. Under general anesthesia, the patient was positioned in lithotomy with a steep Trendelenburg tilt. A nasogastric tube was placed for gastric decompression, and a Foley catheter was inserted for bladder decompression. Pneumoperitoneum was established via a 12-mm umbilical port, with additional working ports placed under direct vision (two 5-mm ports in the right lower quadrant and left iliac fossa, and one 12-mm port in the right upper quadrant).

The inferior mesenteric artery was high-ligated, followed by a medial-to-lateral and lateral-to-medial dissection. The splenic flexure was mobilized to ensure adequate length for a tension-free anastomosis. The rectum was divided using a linear stapler, and a stapled end-to-end colorectal anastomosis was fashioned using a 29-mm circular EEA stapler, with intact donuts confirmed. No proximal covering ileostomy was created. A pelvic drain was placed, and the procedure was completed uneventfully. The postoperative course was uneventful. The patient was mobilized on postoperative day (POD) 1, resumed oral diet on POD 2, and passed stool on POD 3. She was discharged in stable condition on POD 5.

Gross examination revealed a sigmoid mass measuring 7.5 cm in greatest dimension. Microscopy demonstrated a moderately differentiated adenocarcinoma invading the muscularis propria. All resection margins were negative, with no lymphovascular or perineural invasion. Seventeen lymph nodes were examined, all negative for metastasis (0/17). Final pathology demonstrated pT2N0 (Stage I) per the American Joint Committee on Cancer (AJCC) staging manual [5]; no adjuvant chemotherapy was indicated.

The patient was enrolled in a structured surveillance program. Follow-up includes clinical assessment, serum carcinoembryonic antigen (CEA) monitoring, and interval imaging (CT chest/abdomen/pelvis) as per colorectal cancer surveillance guidelines. At her most recent follow-up, four months postoperatively, she remains asymptomatic with no evidence of recurrence.

## Discussion

Adult intussusception is an uncommon condition, accounting for less than 5% of all intussusceptions and <1% of intestinal obstructions [1]. Unlike pediatric cases, which are often idiopathic, adult intussusceptions usually have an organic lead point, with malignancy being the most common cause in colonic disease [2].

Rectal prolapse in adults is generally considered a benign condition, often associated with pelvic floor weakness, constipation, or increased intra-abdominal pressure [3]. However, in rare cases, rectal prolapse may represent the external manifestation of an intussusception driven by a malignant lead point [4-7]. In our patient, the sigmoid adenocarcinoma acted as the pathological lead point. The tumor-bearing sigmoid colon segment (intussusceptum) telescoped into the rectum (intussuscipiens). Peristaltic forces propelled the intussuscepted segment distally until it protruded through the anal canal, where it mimicked rectal prolapse. This mechanism illustrates how an intraluminal malignancy can masquerade as prolapse and highlights the importance of thorough evaluation in such patients.

The relationship between advanced age, colorectal cancer, intussusception, and rectal prolapse is likely multifactorial. Advanced age predisposes to prolapse due to pelvic floor laxity, reduced connective-tissue elasticity, and chronic constipation, while older adults are also at increased risk of colorectal malignancy, which may serve as a lead point for intussusception. When these conditions coexist, the intussuscepted bowel can prolapse externally. This explains why Shahabi et al. (2025) found that most patients with colorectal cancer presenting as rectal prolapse were elderly females with constipation and bleeding [7]. Additional reports by Jurić et al. (2023) [2] and Doita et al. (2024) [8] reinforce this association. Our case was unusual in that the patient was young (39 years), postpartum, and otherwise medically free, yet the same mechanism was operative, underscoring that such presentations can occur outside the typical demographic.

The postpartum state may have contributed to this presentation, as pregnancy and puerperium are associated with constipation, hormonal changes, and pelvic floor alterations that can predispose to prolapse. That said, there is no definitive evidence linking reproductive hormones to colorectal adenocarcinoma pathogenesis; experimental studies suggest that estrogen receptor-β signaling in the colonic mucosa may even have a protective role [9,10].

Another noteworthy point is that both tumor markers (CEA and CA 19-9) were within normal limits despite histologically proven adenocarcinoma. This is consistent with prior evidence that CEA has low sensitivity in stage I-II colorectal cancer and is more useful for prognosis and surveillance in advanced disease, while CA 19-9 lacks specificity and is not recommended for screening or diagnosis of colorectal cancer [11-13]. Thus, normal tumor markers likely reflected the early stage of disease in our patient (pT2N0, Stage I). Oncologic adequacy was achieved with laparoscopic low anterior resection, including high ligation of the inferior mesenteric artery, negative margins, and an adequate lymphadenectomy (17 nodes examined, all negative). These findings yielded a Stage I tumor with a favorable prognosis.

This case adds to the limited but growing evidence that persistent or atypical rectal prolapse should prompt thorough investigation. Cross-sectional imaging and colonoscopy remain essential tools, even in young or postpartum patients, to ensure timely diagnosis of underlying malignancy.

## Conclusions

Colorectal adenocarcinoma rarely presents as rectal prolapse or intussusception, particularly in young postpartum women. This case emphasizes the importance of maintaining a high index of suspicion for malignancy in patients with persistent or atypical prolapse, even when clinical features suggest a benign condition. Prompt evaluation with cross-sectional imaging and colonoscopy is essential for early diagnosis and appropriate oncologic or surgical management.
